# A Vibration Analysis for the Evaluation of Fuel Rail Pressure and Mass Air Flow Sensors on a Diesel Engine: Strategies for Predictive Maintenance

**DOI:** 10.3390/s24051551

**Published:** 2024-02-28

**Authors:** Carlos Mafla-Yépez, Cristina Castejon, Higinio Rubio, Cesar Morales

**Affiliations:** 1 Grupo de Investigación de Ciencias en Red eCIER, Universidad Técnica del Norte, Ibarra 100105, Ecuador; 2 MAQLAB Research Group, Dpto, Ing. Mecánica, Pedro Juan de Lastanosa Reseach Institute, Universidad Carlos III de Madrid, 28911 Leganes, Spain; castejon@ing.uc3m.es (C.C.); hrubio@ing.uc3m.es (H.R.); 3 Grupo de Investigación de Ingeniería Automotriz GIIA, Universidad Técnica del Norte, Ibarra 100105, Ecuador; cfmoralesb@utn.edu.ec

**Keywords:** internal combustion engines (ICEs), mechanical vibrations, Fuel Rail Pressure (FRP), Mas Air Flow (MAF) sensors, predictive maintenance, frequency-domain technique

## Abstract

This research focuses on the analysis of vibration of a compression ignition engine (CIE), specifically examining potential failures in the Fuel Rail Pressure (FRP) and Mass Air Flow (MAF) sensors, which are critical to combustion control. In line with current trends in mechanical system condition monitoring, we are incorporating information from these sensors to monitor engine health. This research proposes a method to validate the correct functioning of these sensors by analysing vibration signals from the engine. The effectiveness of the proposal is confirmed using real data from a Common Rail Direct Injection (CRDi) engine. Simulations using a GT 508 pressure simulator mimic FRP sensor failures and an adjustable potentiometer manipulates the MAF sensor signal. Vibration data from the engine are processed in MATLAB using frequency domain techniques to investigate the vibration response. The results show that the proposal provides a basis for an efficient predictive maintenance strategy for the MEC engine. The early detection of FRP and MAF sensor problems through a vibration analysis improves engine performance and reliability, minimizing downtime and repair costs. This research contributes to the advancement of monitoring and diagnostic techniques in mechanical engines, thereby improving their efficiency and durability.

## 1. Introduction

Internal combustion engine technology has been a mainstay in a wide variety of applications, from ground vehicle propulsion to power generation in industrial plants. Among these, Compression Engines (CEEs) have played a crucial role due to their efficiency and versatility. MEC engines are characterized by their ability to compress air prior to fuel injection, resulting in a highly efficient combustion process [1,2]. This characteristic makes them ideal for a wide range of applications where reliable power and increased energy efficiency are required.

From heavy transport vehicles to power generators, MEC engines are used in sectors as diverse as automotive, marine, aviation, and electric power generation [3]. Their ability to deliver high torque and improved thermal performance makes them a preferred choice in applications requiring a combination of power and efficiency.

Diesel engine vibration is caused by many factors, one of them is the injection system, which can fail for different reasons and affect the dynamic behaviour of the engine; possible causes of injection system failures include premature fuel injection, injection delay, low injection pressure, over-injection, injector clogging, and injection pump failure, so engine vibration indicates the stability and mechanical condition of the engine [4].

Mechanical vibrations are physical phenomena caused by a vibratory motion of a mechanical system or its components, an oscillation of the particles that make up matter, i.e., vibrations are transmitted or propagated from their origin through any physical medium and, except in the case of resonance phenomena, the transmission of vibrations usually involves a damping that depends on the nature of the medium in which they propagate [5].

Several studies of a vibration analysis in diesel engines using biodiesel blends found that they experience less vibrations compared to pure diesel, identifying vibrations at specific frequencies (25 Hz) related to combustion and piston movement; likewise, a 13.7% reduction in vibrations was observed when using biodiesel–diesel blends due to lower cylinder pressure [6,7].

FRP (Fuel Rail Pressure) and MAF (Mass Air Flow) sensors play a critical role in the performance and efficiency of compression ignition engines [8]. Their relevance lies in several fundamental aspects that directly affect the optimal performance of these engines: Fuel Injection Control, Air–Fuel Mixture, Power and Efficiency, Emission Reduction [9].

In the context of Industry 4.0, conventional maintenance strategies, such as corrective and preventive ones, face notable challenges and limitations, such as high costs, mathematically deficient or inaccurate degradation processes, as well as the need to manually extract features. With the increasing adoption of smart manufacturing and the advancement of technologies such as the Internet of Things (IoT), Data Mining (DM), and Artificial Intelligence (AI), as well as semantic representations, the concept of predictive and proactive maintenance emerges. This approach involves performing maintenance interventions only after analytical models predict certain failures or degradations, which offers numerous potential advantages in terms of reliability, safety, and maintenance cost reduction, among other benefits [10,11].

Common Rail, an electronic direct injection system, is an advanced system because it modifies the injection parameters so that the amount of fuel injected varies according to the fuel pressure and flow rate, which contributes to improve combustion by reducing pollutant emissions and consumption, as well as increasing power and reducing noise and vibrations, compared to conventional diesel engines [12,13].

The improvement in combustion efficiency in diesel engines is influenced by several factors, such as the fuel injection characteristics, which include the number of injections, the time intervals between injections, the amount of fuel injected, and the injection pressure. Modifying these particularities directly impacts the vibrations experienced by the engine [14]. To analyse these vibrations, different signal processing methods have been applied, such as time-domain, frequency-domain, and time-frequency transforms (TFRs) and wavelet transforms [15,16]. TFR transforms are especially valuable for structural integrity monitoring in vibrating systems. These approaches have wide utilities in vibration troubleshooting and diagnostics of rotating machinery [17]. For example, the short-time Fourier transform (STFT) is used to detect vibrations originating from various sources in an engine and to identify combustion-related irregularities in cylinders.

Various factors, such as combustion and knocking phenomena, are the main sources of vibration and noise in engine operation. In the context of diesel engines, as the ignition delay increases, the knocking phenomenon intensifies compared to spark ignition engines. In this situation, knocking causes annoying noise and vibration emissions, resulting in piston crown degradation and damage to engine components [18].

The vibration amplitude in an internal combustion engine is a crucial indicator of engine condition and performance [19]. It is the maximum magnitude of engine vibrations and can be caused by several factors, including fuel combustion, piston motion, and interactions between engine components [20]. Measuring and analysing vibrational amplitude can be useful for diagnosing and predicting maintenance of internal combustion engines. The amplitude of these vibrations can be influenced by the condition of engine components, fuel quality, and operating conditions [21].

Online monitoring of vibration characteristics in both the time domain and frequency domain ensures more efficient, reliable, and adaptive processing machinery, which contributes to accurate production and higher product quality by enabling the early detection of machinery failures [22].

A vibration analysis is mainly used in the monitoring of rotating machines. Traditionally, in a time domain analysis, the time evolution of statistical parameters such as RMS, kurtosis, or peak value, among others, was studied. In the frequency domain, studies of frequency spectra through the application of the Fourier transform allow for detecting the type and severity of failure of some critical elements of the machine (such as bearings, shafts, or gears, among others) because their failure frequencies are widely known. However, it is not always possible to detect them in service [23].

The reasons behind vibration in diesel engines are diverse and complicated, encompassing factors such as combustion problems, mechanical imbalances, and resonances in the structure [24]. Engine block vibration is mainly attributed to variations in combustion pressure in the cylinders. During diesel engine operation, inconsistencies in combustion can lead to in-cylinder pressure fluctuations, which, in turn, cause periodic vibrations in the engine block [25].

Research conducted on four-stroke marine diesel engines on a test bed revealed that the FFT analysis confirmed the presence of frequencies that were sensitive to changes in injector opening pressure only in the initial signals. However, vibrations of the crankshaft and piston system could mask these frequencies in subsequent measurements. By changing the mechanical injector pressure during engine operation, unique data were collected. The analysis confirms that vibration methods can be used to diagnose malfunctions in marine piston engine injectors [26].

Çalık [27] compared the vibration results of engines using different fuel additives. The study found that an increase in vibration was observed with hydrogen–diesel and hydrogen–biodiesel blends as engine speed increased. However, according to Boyukdipi’s study [28], engine vibration decreases at high speeds due to a decrease in combustion efficiency and in-cylinder pressure caused by the lack of oxygen content of the NH3 additive. The study concludes that the type of fuel additive can directly affect engine vibration as engine speed varies.

In this era of focus on sustainability and energy efficiency, understanding and optimizing MEC engines have become critical. The research and analysis of critical components such as FRP (Fuel Rail Pressure) and MAF (Mass Air Flow) sensors play an essential role in improving the performance and reliability of these engines. Therefore, exploring predictive maintenance strategies for MEC engines through a vibration analysis is of great importance today.

## 2. Materials and Methods

**Figure 1:**
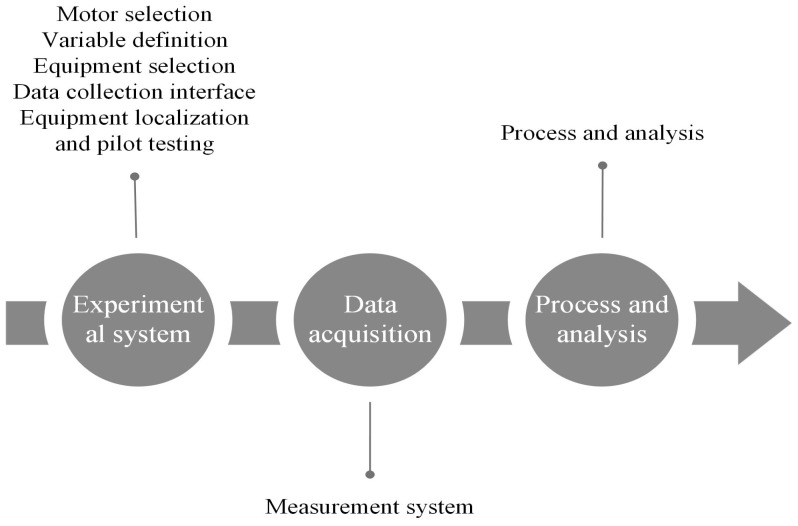
Methodology of the experiment.

Predictive maintenance, specifically condition monitoring, is a crucial option due to its ability to perform real-time diagnostics of vehicles and anticipate potential faults, enabling corrective measures to be taken prior to critical engine situations. It is important to note that several tasks are associated with preventive maintenance, although this study focuses on evaluating vibrations. The methodology used is illustrated in Figure 1. The experiment commenced with the selection of the engine as the starting point, followed by defining the variables to be studied. After that, the sensors were selected, and their placement in both the engine and the acquisition card was determined, ensuring suitable choices for the analysis. A specific interface was then developed for data acquisition, taking into account various characteristics, including instances of good condition and situations with faults. Finally, a processing procedure was carried out on these data for a subsequent analysis.

As part of this study, we gathered experimental data using a Mazda BT50 4-cylinder CRDi electronic diesel engine (Hiroshima, Japan). Technical term abbreviations will be explained when first introduced. The engine was mounted on an experimental test rig (please see the included photo). This particular engine has a power output of 105 kW and is equipped with 16 valves. Notably, it features a common rail injection system incorporating a rail pressure sensor (FRP), as well as an air flow sensor (MAF) of a comparable nature, both of which are Bosch branded (Gerlingen, Germany). This study focuses on identifying the most common and recurrent faults in the sensors under investigation. Sensors are responsible for converting mechanical phenomena into electronic signals, and any failure can affect their output. To gain a better understanding of these variations, simulations were conducted for different operating states. The technical features of the engine and its fuel injection system were crucial for this research’s progress since they offer vital data for analysing and evaluating its performance regarding vibrations and other critical parameters. Table 1 displays the most significant engine features from its datasheet.

To gather data from the testbench, we utilised an industrial accelerometer that is part of the ICP 603 C01 series of the company PBC Piezoelectronic (Depew, NY, USA). This sensor is [29] certified and was selected due to its exceptional long-term stability, capacity to endure fluctuations in temperature, high resolution, and robustness in both low- and high-frequency measurements, making it an advantageous choice for this study. A specially designed magnet was applied for the attachment of the sensor in order to guarantee a robust and secure attachment. This decision impacted considerably on the accuracy and reliability of the measurements taken during this study.

The vibration sensor was connected to the computer via USB, employing the National Instruments DAQ 9250 (Austin, TX, USA) data acquisition board. This board requires no additional configuration and draws its power directly through the USB bus, removing the necessity for an external power supply. Its primary purpose is to convert physical signals collected by the sensor into electrical signals that can be graphically presented on the computer, thereby enabling an efficient and precise vibration data acquisition and analysis.

LabVIEW SP1, 2020 software from National Instrument (Austin, TX, USA) was utilised for collecting the data, with specific programming, whilst the vibration data obtained were processed with MATLAB R2021a software from MathWorks, Inc. (Natick, MA, USA). This amalgamation of tools enabled proficient data management and a comprehensive data analysis.

### 2.1. Experimental Test Design

In an experimental procedure, researchers distinguish two types of variables: controlled and uncontrolled. Controlled variables are maintained at specific levels, such as the engine’s operating temperature, set at 60 °C, and the engine load, set at the minimum operating condition (i.e., idling). In contrast, variables that are not manipulable during the experiment, including atmospheric pressure, ambient temperature, and relative humidity, are referred to as uncontrolled. For consistency, it is crucial to distinguish between the two.

This study employed identical accelerometers installed in accordance with [30] guidelines. One sensor was horizontally positioned at the top centre of the engine block (in the axial direction of the crankshaft), whereas the other was located on the valve cover toward the cylinder head (in the direction of piston advancement). For each test, a thorough check was conducted to ensure that both the accelerometers and cables were installed firmly to prevent signal distortion from the accelerometer to the acquisition board.

Combustion is the stage where the engine generates its primary energy for operation, leading to the emission of considerable vibrations caused by the force that drives the piston toward its bottom dead centre [31]. Hence, to prevent any noise from the distribution and gearbox, a central sampling point near the combustion chamber was strategically identified and located.

**Figure 2:**
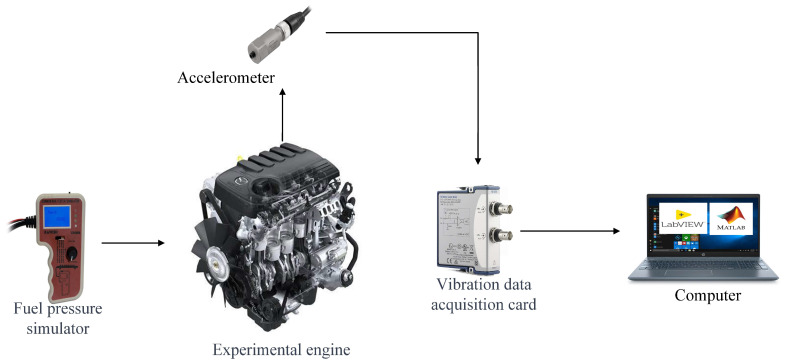
Experimental testbench and measurement chain.

Please refer to Figure 2 for the engine vibration monitoring process. The accelerometers are positioned in accordance with ISO-5348 regulations at designated locations. The necessary preparations for acquiring vibration measurements were then made. Once ready, with the engine in operation, LabVIEW-designed software was employed to collect the vibration data. Subsequently, the collected data were analysed using MATLAB software.

During the process of the data analysis, the Fourier transform was used to analyse the signals and describe them based on the outcome of each test carried out. This methodology offers a comprehensive comprehension of the engine vibrations and allows for the recognition of important patterns and characteristics that are crucial for an engine performance diagnosis and evaluation. The implementation of these techniques and tools promotes a meticulous and precise examination of the engine vibrations in question.

The experiment was conducted in three distinct scenarios:

- scenario one portrays ideal engine operation (normal operation).
- scenario two simulates an MAF sensor failure (failure 1, F1), with subcategories
  - incipient fault (F1.1);
  - moderate fault (F1.2).
- The third simulates a failure in the FRP sensor (failure 2, F2).

All scenarios were conducted under identical operational conditions, with the working temperature at 60 °C and the engine idling.

It should be noted that 200 samples were taken for each scenario, and the tests were carried out on different days to ensure the robustness and reliability of the obtained results.

### 2.2. Simulation of MAF Sensor Failure

In the context of this engine, a 5-wire MAF sensor inclusive of 2 wires for the temperature sensor was employed, and a potentiometer was utilised to modify the sensor’s signal. To ensure correct adjustment, each wire was independently identified (1—power, 2—signal, 3—ground, 4-5—temperature), and jumper connections were made for wires 1 and 3. The signal wire (2) was connected to the potentiometer so that voltage could be adjusted to the required values. A value of 2.1 V was established for typical operation regarding an idle engine, while the fault sensor simulation was altered to 2.5 V (incipient fault) and 3.5 V (moderated fault). This signal modification mimics a fault in air admission. To ensure precise and consistent results, the test was replicated in four separate simulations.

### 2.3. FRP Sensor Failure Simulation

To calibrate the pressure in the Common Rail, we utilised the CR508S Common Rail Pressure Tester from Hubei Aovite Automotive Machinery Co., Ltd. (Wuhan, China). Technical term abbreviations are explained upon first use. As the engine features a Bosch high-pressure pump, we employed a connector specially designed by Bosch for the corresponding unit. This connector, also known as a jack, is directly wired to the FRP sensor socket, with its other end connected to the sensor itself. The language is clear, objective, and follows a formal register, adhering to grammatical correctness and balanced writing. The sensor measured 38.70 MPa under normal operating conditions, while the failure simulation showed 40.70 MPa (incipient fault) and 55.70 MPa (severe fault). To guarantee the precision and coherence of the results concerning the injection system pressure, the configuration was tested using four different simulations.

### 2.4. Vibration Data Acquisition 

**Figure 3:**
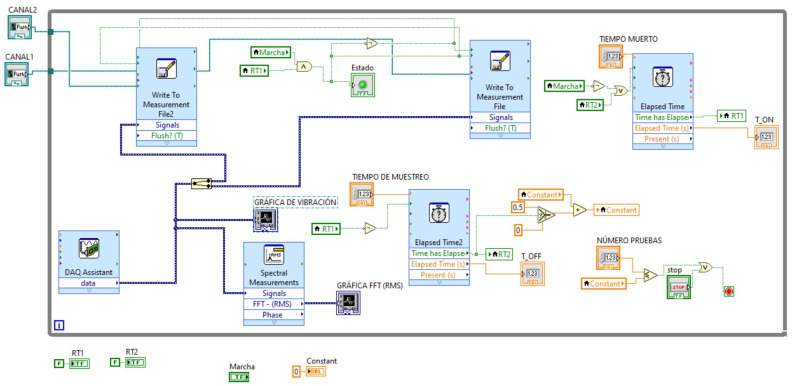
Programming vibration data acquisition.

LabVIEW software was utilized to develop a bespoke research programme to obtain vibrational data from the testbench. The programme enables the automated collection of certain data sets based on the established acquisition parameters in this study (refer to Table 2), which include the sampling frequency, time interval between captures, number of samples, and location for information storage, amongst others. The schedule of the LabVIEW project is shown in Figure 3.

When obtaining and processing signals, it is crucial to consider various parameters that determine the data’s quality. Initially, a sampling frequency (Fs) of 8.123 Hz was established for all experiments, which should align with the accelerometer’s characteristics in use. Additionally, it is essential to specify the number of points (N) to acquire during each measurement. To prevent the zero-padding effect that may introduce erroneous data to the signals, it is essential that this figure is a power of two [32]. The data acquisition parameters are collated in Table 2.

Since the lowest frequency recorded during engine operation is considered, which is set at 10 Hz, and at least 100 cycles are sought to be captured in each signal, a minimum duration of 8.02 s is set for each signal. The closest power of two that meets these criteria is 216, equivalent to 65,536 points (N). Thus, using the following equation, the duration of each vibrational signal can be accurately calculated:

(1) $$T=\frac{N}{Fs}=\frac{64.000}{8.162}=8.02\;s$$

This approach ensures that the sampling rate is appropriate for the sensor and that the number of points acquired allows an accurate representation of the signals, avoiding zero-filling problems. It also ensures that sufficient information is captured for the duration of the signal to meet data acquisition objectives.

### 2.5. Vibration Data Processing 

Out of the many samples gathered in each experiment—a total of 200 per trial, with each one producing 64,000 values—it was decided that a more efficient data analysis would be performed by randomly selecting only 30. It is crucial to note that, during this selection process, the initial and final 50 samples of each trial were excluded. This approach involves selecting 30 samples from each trial to achieve a more precise and representative data analysis, resulting in significant and consistent findings. Removing the initial and final points of each trial reduces the impact of unstable or irregular initial and final conditions that can differ from the stable conditions during the experiment. Thus, this approach ensures dependable and precise outcomes for the executed analysis by reducing potential biases introduced at the start and end.

## 3. Results

The analysis centred on tabulating the data concerning the motor’s harmonics in each simulation. This facilitated an assessment of the motor’s behaviour under each fault condition. It is worth noting that every sensor’s data were analysed separately in the vertical and horizontal directions to identify patterns and trends and uncover potential variances between simulations. The average data from the five scenarios were subsequently calculated and used to determine the corresponding operating ranges. Following this, the data obtained were subjected to a frequential analysis, which was conducted as separate studies for each accelerometer.

### 3.1. Vertical Sensor Analysis (Valve Cover)

**Figure 4:**
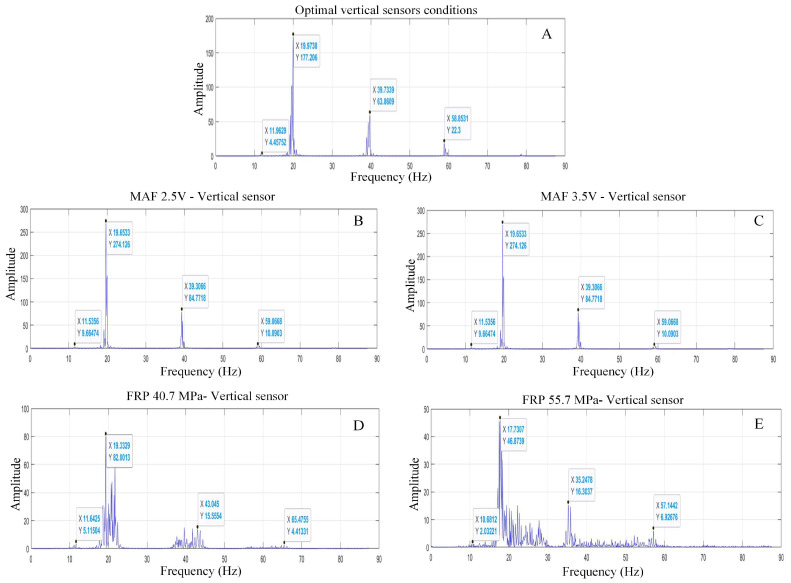
Motor frequency spectra for different working conditions, including faults and sensor simulations ((**A**): normal operation; fault in MAF sensor—(**B**): incipient, (**C**): moderated; fault in FRP sensor—(**D**): moderated, (**E**): severe).

The fuel–air combination within the combustion chamber undergoes rapid expansion, thus generating a substantial amount of pressure. Consequently, this pressure propels the pistons, creating force and movement, which are then conducted to the crankshaft and the entire engine system. The largest peak resulting from the combustion can be inferred from the position and features of the vertical accelerometer, as illustrated in Figure 4. Furthermore, this evidence is corroborated with the horizontal accelerometer, demonstrating a larger amplitude at the corresponding frequency of 20 Hz.

Figure 4 shows the characteristics of the spectrum generated with the motor under ideal conditions (Figure 4A) and with the respective fault simulations (Figure 4B–E). In Figure 4, four peaks of frequency appear, the first being 11.75 Hz corresponding to the regime velocity (established to 700 rpm in the test), the second being 19.97 Hz corresponding to the combustion process [31], and peaks 3 and 4 are the harmonics of the previous one. In the rest of the plots represented in Figure 4, the four peaks with significantly different amplitudes are also clearly identified. It is worth mentioning that this graphical representation is one of multiple tests, and consistent behaviour is observed in all the samples collected.

The spectra offer an extensive understanding of the motor’s conduct under varied circumstances, assisting in the comprehension of potential concerns and performance fluctuations.

Table 3 shows the frequency and amplitude ranges of motor behaviour in various simulations of the investigation. This provides a lucid outlook of the operating ranges under different arrangements.

This study analysed engine performance under various fault simulations and identified amplitude ranges corresponding to the second peak, which indicates the predominant combustion phase. The engine is considered to be in optimum condition when peak 2 is at 19.72 Hz. In the MAF sensor simulation with a 2.5 V signal, there is a slight drop of 0.20% relative to the average, indicating moderate damage. However, when the MAF signal is 3.5 V, the range of the average is reduced by 5%, indicating a more significant deterioration.

Simulations of the FRP sensor indicate significant differences. At 40.7 MPa, the mean value range decreases by 7%, indicating severe damage. The situation becomes critical at a pressure of 55.7 MPa, where the mean value decreases by 10%, causing severe vibrations.

For F3, under optimal conditions, the amplitudes are always close to the mean of 39.40. With an MAF sensor signal of 3.5 V, the average amplitude drops by 0.2%, which does not indicate any significant damage. However, the situation changes significantly when considering the FRP sensor. At a pressure of 40.7 MPa, the average amplitude range varies by 6%, indicating significant instability and potential engine damage. The situation worsens as the pressure rises to 55.7 MPa, where the mean range drops by 9%, representing the most critical state of the engine.

Under optimal conditions, the F2 and F3 frequencies remain stable. This study shows that the MAF sensor failure simulations are consistently within ranges close to the ideal state. The engine is considered to be operating optimally with an average of 19.71 for the second and 39.42 for the third harmonic frequencies. However, instability is noticeable when these values drop to 17 and 36, respectively, indicating a serious problem. The analysis concludes that the engine is unable to maintain idle speed and experiences significant acceleration and deceleration, indicating a serious mechanical problem.

Table 3 displays the peaks of frequency 2 (F2) based on the standard deviation. The data obtained from different operating conditions are acceptable as their values are lower than 1.71, which is the reference value for their condition. This indicates that there is no fault in the MAF sensor. There is no significant difference between 2.5 V and 3.5 V. Similarly, it has been verified that the engine operation does not change with a difference of 0.10% and 2.44%, respectively. Therefore, it is not possible to detect any difference in motor operation. Changes in engine operation were observed for the FRP sensor failures at 40.7 MPa and 55.7 MPa. The mean frequencies (refer to Table 3) also show a slight variation of 4.72% and 10.60%, respectively, indicating an engine failure. Regarding harmonic 2, the engine failures studied (MAF 2.5 V and 3.5 V, FRP 40.7 MPa and 55.7 MPa) show the same trend. The reference value is 234.85, which corresponds to the optimum conditions. The operation varies by 3.76%, 7.93%, 16.93%, and 74.02%, respectively, indicating that the most significant damage is to the FRP sensor.

**Figure 5:**
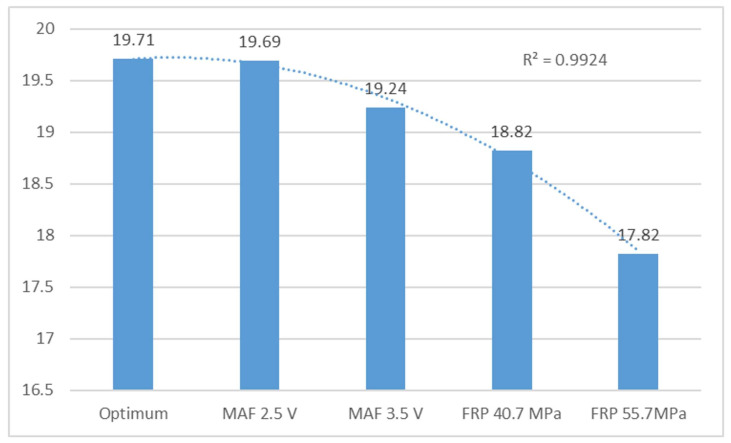
Averaged frequency peaks 2 (F2) in Hz of the vertical sensor.

Figure 5 shows the frequency of peak 2 behaviour, with an R^2^ value of 0.99, indicating good values as it is close to 1. The trend line suggests that the values are favourable. Any variation in frequency indicates a change in engine speed.

**Figure 6:**
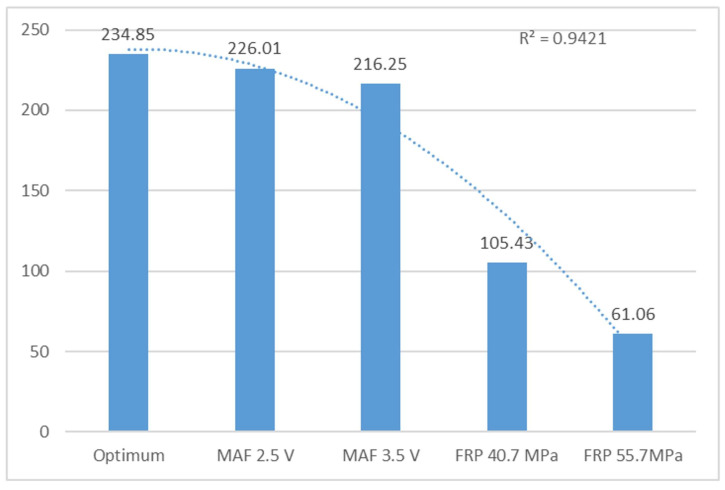
Average ammonia amplitude level from vertical sensor peak 2 in V.

Figure 6 displays the harmonic of peak 2, indicating significant changes in engine operation. The FRP sensor failures cause notable variations in the harmonics and engine performance. Additionally, the R^2^ value is close to 1, with a value of 0.94.

**Figure 7:**
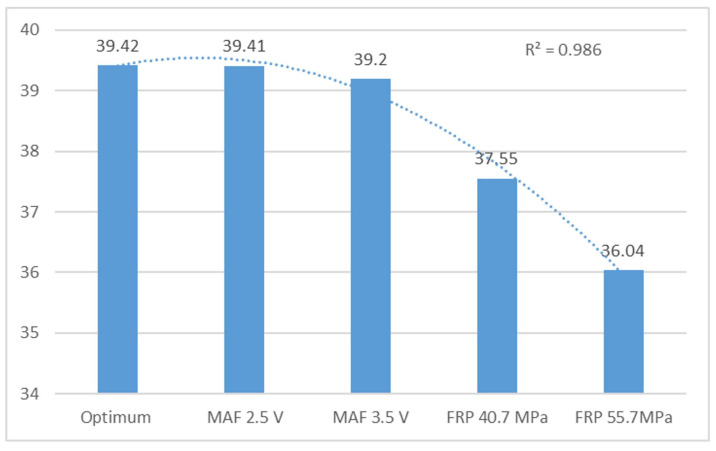
Averaged frequency peaks 3 (F3) from vertical sensor in Hz.

Table 3 presents frequency peak 3 (F3) and its harmonic, which serve as a reference for optimal conditions at 39.42 Hz. MAF faults (2.5 V and 3.5 V) vary by 0.025% and 0.55%, respectively, at this frequency, while FRP sensor faults (40.7 MPa and 55.7 MPa) exhibit a variation of 4.75% and 8.50%, respectively. The motor experiences more significant failure with FRP faults, causing variations in speed and frequency. However, the harmonic trend remains unchanged. Changes in operation based on the studied faults (MAF 2.5 V and 3.5 V and FRP 40.7 MPa and 55.7 MPa) revealed optimum operating conditions, resulting in variations of 14.22%, 20.81%, 65.70%, and 78.66%, respectively. Frequency peaks 2 and 3 are similar. Figure 7 illustrates the behaviour of frequency peaks as they decrease depending on the analysed fault. The FRP 55.7 MPa exhibits the most significant frequency variation due to sensor failure, which alters engine speed. The R^2^ value of 0.98 suggests that the independent variables of the model account for the variations in the dependent variable.

**Figure 8:**
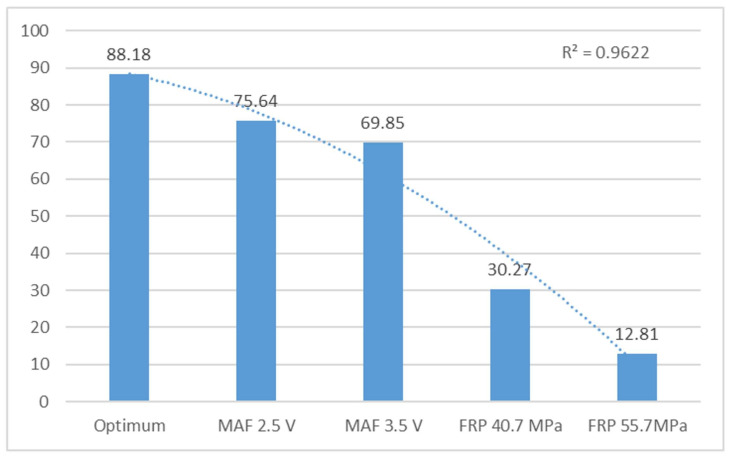
Average amplitude of peak harmonics 3 of the vertical sensor in V.

Figure 8 displays the harmonics in the various engine states. The FRP 55.7 MPa sensor failure exhibits a greater variation, with a coefficient of determination R^2^ of 0.96, which is very close to 1.

### 3.2. Horizontal Sensor Analysis (Cylinder Block)

**Figure 9:**
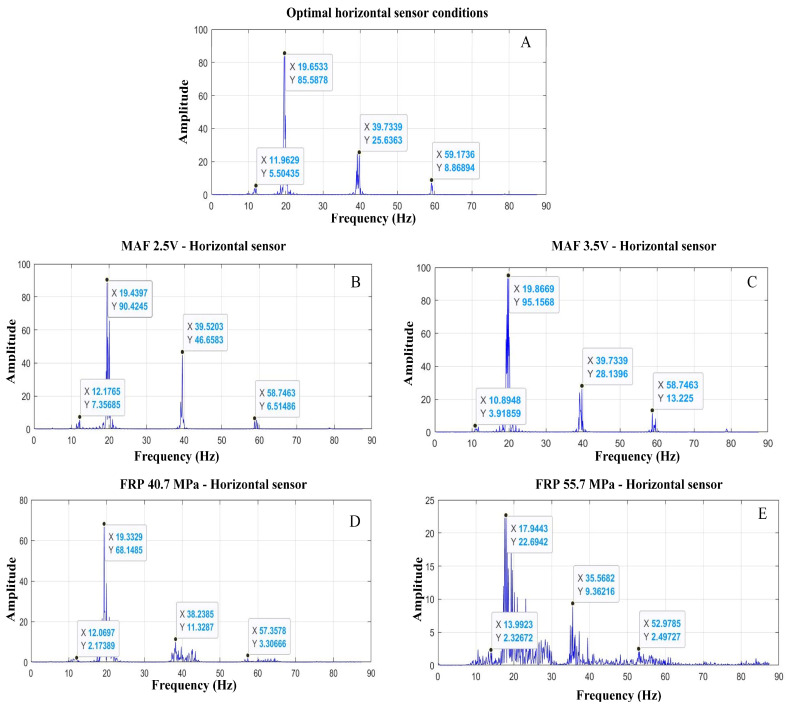
Motor spectra: ideal conditions, sensor variations, and failures in different operating states with a horizontal sensor ((**A**): normal operation; fault in MAF sensor—(**B**): incipient, (**C**): moderated; fault in FRP sensor—(**D**): moderate, (**E**): severe).

Collecting vibration data from the cylinder block facilitates the identification of engine conditions based on combustion. Figure 9 displays the spectra collected at various engine operating states.

Similarly, four frequency peaks are identified, and F2 and F3 will undergo scrutiny as with the previous accelerometer. Additionally, a repetition of frequency is observed from the second peak forward, with an average value of 20 Hz. In all tests, it is observed that the higher frequencies (F3 and F4) are consistently repeated during the motor test in its optimal state (Figure 9A). This graphical depiction of vibrations presents distinct frequencies without excess noise that could impede the measurement’s accuracy.

The spectra present a thorough outlook of the engine’s performance, portraying its responses to sensor fluctuations and virtual malfunctions under diverse circumstances.

Table 4 presented below highlights the frequency and amplitude ranges of motor behaviour in the different research simulations, enabling the identification of motor intervals operating with the varying settings.

In the analysis of the data from the vertical sensor, a coherent trend in its behaviour is highlighted. Focusing on F2, it is important to note that these results are based on multiple signals acquired in various tests (30 samples).

The text examines the effects of simulated failures in Mass Air Flow (MAF) and Fuel Rail Pressure (FRP) sensors on engine performance. The simulations of the MAF sensor, ranging from 2.5 V to 3.5 V, had a negligible impact on engine performance, with mean data fluctuations of only 0.1% and 0.2%, respectively. However, the study found that faulty FRP sensors can significantly impact engine performance due to disrupted fuel supply conditions. The mean data fluctuations were 6% for FRP 40.7 MPa and 8% for FRP 55.7 MPa. This study concludes that sensor failures, particularly in the FRP sensor, can have a catastrophic impact on engine behaviour. The text already meets the desired characteristics and is free from errors. Therefore, no changes are necessary.

Table 4 presents a frequency range that corresponds to harmonics 1 and 2 in all simulations undertaken. This arrangement of data aids in comprehending and analysing frequency behaviour when obtaining the vibration spectra. The integration of these frequency ranges is a valuable resource for precisely contextualizing and evaluating the characteristics of vibration signals in each study scenario. This significantly aids the interpretation and comprehension of research findings, providing a more comprehensive and detailed insight into the variation of harmonic frequencies based on diverse operational conditions and simulations. The precise representation of this information within the tabulated format serves as a critical resource for a subsequent analysis and aids in the progression of knowledge in the domain of machinery and engine vibration, facilitating prudent diagnostic and maintenance determinations.

In the context of analysing the frequency of vibrational data, a consistent pattern is observed in most failure simulations, apart from the FRP sensor at 55.7 MPa, where a significant change occurs. This alteration in frequency can be attributed to a change in engine behaviours and a decrease in idle speed. The sensitivity of the frequency is emphasized as a crucial indicator of engine operating conditions.

When examining the second harmonic, it is evident that, given ideal conditions, the mean frequencies maintain a range of 19.71. This discovery implies that the second harmonic is notably stable when subjected to standard operating conditions. Similarly, for the third harmonic, the mean frequencies sustain consistency, remaining at 39.46.

Noticeable variations are evident in simulations of the FRP sensor, primarily influenced by idle speed changes. In the case of the MAF sensor, there are also variations in frequencies, although they are not as substantial as those observed with the FRP. Such variations could point to potential system faults and signify the significance of closely monitoring frequencies to identify and remedy engine issues.

A frequency analysis of vibration data provides important insights into motor behaviour and fault detection, illuminating stability during regular operations and sensitivity to variations when faults or significant changes in motor operation arise. This understanding is vital for the efficient diagnosis and maintenance of engines in diverse applications.

Table 4 examines the behaviour of frequency peaks 2 and 3, as well as their harmonics, in a sensor. Frequency 2 (F2) is analysed with a reference of 19.72 Hz. Variations are observed for different operating states (MAF 2.5 V–3.5 V and FRP 40.70 MPa–55.7 MPa), with percentage variations of 0.10%, 0.15%, 4.39%, and 9.32%. The harmonics data are affected by motor faults. The optimal state (136.62) is the reference for faults, with variations of 2.04%, 6.14%, 33.58%, and 75.66%.

Table 4 presents the frequency peaks and harmonics 3 (F3) data obtained under optimal conditions for analysing the motor’s behaviour with specific operating conditions. The frequency peaks exhibit differences of 0.28%, 7.88%, 10.39%, and 14.99%, while the harmonics show differences of 5.93%, 7.34%, 80.12%, and 82.98%. It is worth noting that greater data variation occurs when the rail pressure sensor fails.

**Figure 10:**
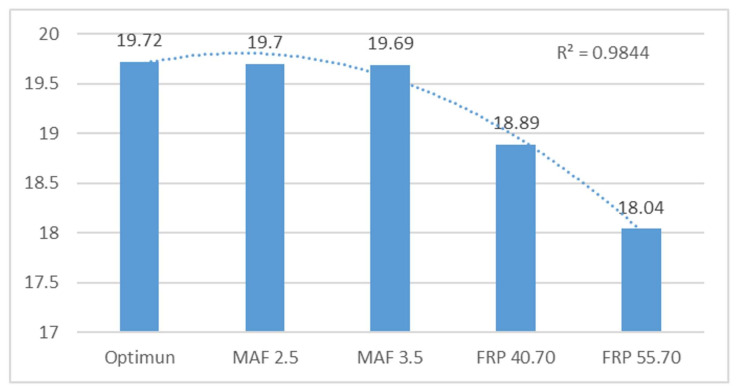
Averaged peak frequency 2 (F2) in Hz of the horizontal sensor.

Figure 10 shows the frequency peaks of the MAF and FRP sensors in optimal and failed states. The MAF sensor shows no significant variation, while the FRP sensor data change drastically. An R^2^ value of 0.98 was obtained, indicating a good correlation with the data.

**Figure 11:**
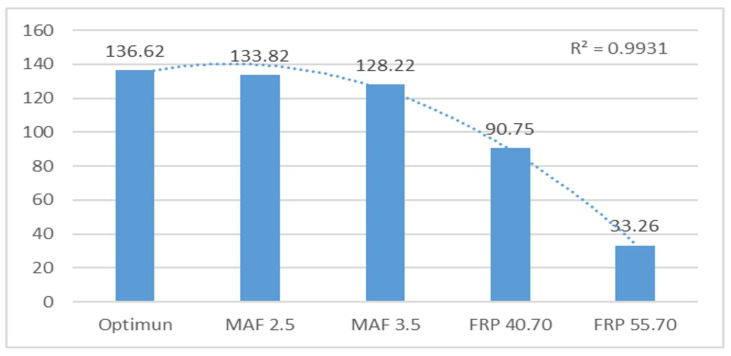
Ammonia amplitude level from vertical sensor peak 2 in V of the horizontal sensor.

Figure 11 analyses harmonics 2, revealing a significant difference in the failures of the FRP sensor due to fuel variation in the rail, which affects its operation. The obtained R^2^ value of 0.99 is close to 1.

**Figure 12:**
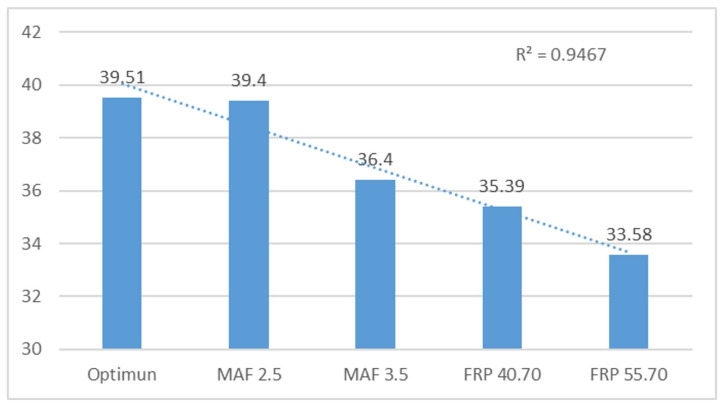
Averaged frequency peaks 3 (F3) from horizontal sensor in Hz.

Figure 12 shows the analysis of frequency peaks and their respective harmonics. The behaviour indicates more variation with failures in the FRP sensor and with the MAF 3.5 V sensor, while there is a variation with the MAF 2.5 failure and in the optimum state. An R^2^ value of 0.94 was obtained, which is acceptable.

**Figure 13:**
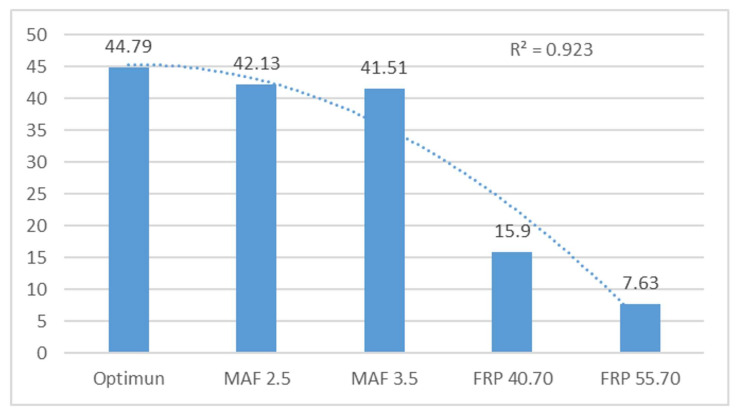
Averaged peak harmonics 3 of the horizontal sensor in V.

Figure 13 analyses the harmonics of peak 3. A significant difference is observed with the failures of the FRP sensor, while the other engine operating conditions remain relatively constant. An R^2^ value of 0.92 was obtained.

## 4. Conclusions

From this exhaustive analysis of the vibration signals in the engine under various conditions and fault simulations, significant conclusions can be drawn. Spectra of normal operation reveal that the dynamic behaviour of the machine presents two main frequencies corresponding with the regime velocity (F1) and the combustion process (F2), highlighting the two harmonics of F2 (F3 and F4). The simulations with the MAF sensor, although they did not show significant changes in frequencies, revealed a negative trend in amplitudes, especially when the MAF signal reached 2.5 V and 3.5 V. These variations in amplitudes with respect to the optimal state indicate anomalies.

On the other hand, simulations with the FRP sensor exhibited immediate changes in the motor, indicating a clear failure in the system. At a pressure of 40.7 MPa, considerably lower amplitudes were observed in both sensors and a wide variability in frequencies, denoting a clear instability in the engine and its inability to maintain a constant idle speed. However, the most critical simulation was evident in the case of the FRP sensor at a pressure of 55.7 MPa. Here, the motor operated in a significantly reduced range compared to the ideal condition, with amplitudes between 53.48 and 68.31 at the vertical sensor and 28.09 and 39.54 at the horizontal sensor. In addition, the frequencies were reduced to a range of 17.32 Hz–18.09 Hz. This situation represented a high risk of failure due to the remarkable instability in the engine behaviour, characterized by fluctuations in the idle speed.

Ultimately, these findings highlight the critical importance of maintaining optimal sensor conditions and FRP sensor pressure to ensure stable and efficient engine operation and underscore the need for rigorous monitoring to detect and address potential failures before they become serious problems.

With the use of both vertical and horizontal sensors, it was possible to correlate the amplitude peaks and the combustion frequency by means of their specific location on the valve cover and cylinder block. The horizontal sensor proved to be more effective in capturing a greater number of frequency peaks, focusing on the second one, which coincided with the one recorded using the vertical sensor at a frequency of 20 Hz. This finding made it possible to determine that this frequency peak is directly related to the combustion process. In the analysis of the faults presented, it was concluded that the vibration data obtained with the horizontal sensor facilitate a more precise appreciation of the changes in the engine behaviour, facilitating a more efficient diagnosis.

This study examines the impact of sensor failures on engine performance, specifically focusing on Mass Air Flow (MAF) and Fuel Rail Pressure (FRP) sensors. It concludes that FRP sensor failures have a significant impact on engine performance, while MAF sensor failures do not. Additionally, this study analyses vibration data frequency, which can aid in diagnosing system faults. The variations in these frequencies under different engine conditions highlight their sensitivity and the importance of closely monitoring them. The importance of sensor health and a frequency analysis in fault detection is highlighted by the research, which contributes to the theoretical understanding of machinery and engine vibration. This knowledge is crucial for developing efficient diagnostic and maintenance strategies for various engine applications.

## Figures and Tables

**Table 1:** Technical characteristics of the engine.

Item	2.5 Mazda-CD Engine
Engine	In-line 4-cylinder, DOHC 16-valve, turbo-charged
Displacement	2499 cm^3^
Diameter × stroke	93 × 92 mm
Compression index	18.0:1
Max. energy	105 kW (143 hp) @ 3500 rpm
Max. torque	330 Nm at 1800 rpm
Emission standard	EURO IV

**Table 2:** Data Acquisition Parameters.

Sampling Frequency (KHz)	8.123
Number of data values per sample	64,000
Total samples/scenario	200
Number of accelerometers	2

**Table 3:** Amplitude and frequency data for different motor operating states (vertical sensor).

Simulation	Peak Frequency 2 (F2)		Harmonic 2	
	Mean	Standard deviation	Mean	Standard deviation
Optimum conditions	19.71	0.047	234.85	6.72
MAF 2.5 V	19.69	0.02	226.01	6.45
MAF 3.5 V	19.24	0.05	216.25	9.06
FRP 40.7 MPa	18.82	0.75	105.43	4
FRP 55.7 MPa	17.82	0.29	61.06	6.64
	**Peak Frequency 3 (F3)**		**Harmonic 3**	
Optimum conditions	39.42	0.032	88.18	7.28
MAF 2.5 V	39.41	0.05	75.64	6.78
MAF 3.5 V	39.20	0.33	69.85	4.94
FRP 40.7 MPa	37.55	1.57	30.27	3.75
FRP 55.7 MPa	36.04	0.78	12.81	1.85

**Table 4:** Amplitudes and frequency recorded at different engine operating states (using a horizontal sensor).

Simulation	Peak Frequency 2 (F2)		Harmonic 2	
	Mean	Standard deviation	Mean	Standard deviation
Optimum conditions	19.72	0.05	136.62	2.69
MAF 2.5 V	19.70	0.04	133.84	5.48
MAF 3.5 V	19.69	0.04	128.22	5.65
FRP 40.7 MPa	18.89	0.74	90.75	6
FRP 55.7 MPa	18.04	0.05	33.26	4,61
	**Peak Frequency 3 (F3)**		**Harmonic 3**	
Optimum conditions	39.51	0.17	44.79	3.07
MAF 2.5 V	39.4	2.94	42.13	4.70
MAF 3.5 V	36.4	0.02	41.51	4.09
FRP 40.7 MPa	35.39	1.68	8.90	2.77
FRP 55.7 MPa	33.58	0.76	7.63	1.37

## Data Availability

Data are contained within the article.
